# Evaluation of Warm Rubberized Stone Mastic Asphalt Mixtures through the Marshall and Gyratory Compactors

**DOI:** 10.3390/ma13020265

**Published:** 2020-01-07

**Authors:** Juan Gallego, Ana María Rodríguez-Alloza, Leticia Saiz-Rodríguez

**Affiliations:** 1 Departamento de Ingeniería del Transporte, Urbanismo y Territorio, Universidad Politécnica de Madrid (UPM), C/ Profesor Aranguren 3, 28040 Madrid, Spain; juan.gallego@upm.es; 2 Departamento de Ingeniería Civil: Construcción, Infraestructura y Transporte, Universidad Politécnica de Madrid (UPM), C/ Alfonso XII 3, 28014 Madrid, Spain; 3 SIGNUS Ecovalor S.L: C/ Caleruega 102 5°, 28033 Madrid, Spain; lsaiz@signus.es

**Keywords:** stone mastic asphalt, warm mix asphalt, rubberized asphalt, gyratory compactor, Marshall compactor

## Abstract

Stone mastic asphalt (SMA) mixtures exhibit excellent behaviour; they are highly resistant to reflective cracking and permanent deformation, as well as providing the wearing surface with an optimal texture. However, the production and compaction temperatures are similar to conventional mixtures, which means that there is a significant consumption of energy, as well as greenhouse gas emissions. Warm mix asphalt (WMA) technology, which has been developed over the last few years, might allow lower temperatures without compromising the mechanical behaviour of the mixtures. Also, over the last few decades, rubberized asphalt has proved to be effective in improving the performance and being environmentally suitable, but it requires higher production temperatures than conventional mixtures. In this study, several tests were performed to evaluate the effect of a chemical WMA additive on the compactability and water sensitivity of rubberized SMA mixtures with both the Marshall and the gyratory compactor. The investigation has shown that the gyratory compactor is more suitable for studying compactability and the water sensitivity of rubberized SMA with WMA additives.

## 1. Introduction

Stone mastic asphalt (SMA) mixtures offer increased durability, are highly resistant to permanent deformation and reflective cracking and provide a wearing surface with a proper texture. These characteristics are based on the high content of binder that is achieved with a good mineral skeleton, a high proportion of filler and the incorporation of additives, especially cellulose fibres, which allow for a higher content of binder without risk of binder drainage.

Polyamide fibres and fibres, including those coming from the recycling of cardboard cellulose, are the most common stabilizing additives for the prevention of the drain-down of the binder [1]. These types of cellulose fibres from recycling give the SMA mixture an excellent performance against drain-down, plastic deformation or water susceptibility [2]. However, if the fibres function as drain-down inhibitors, a modifier such as Styrene-Butadiene-Styrene (SBS) is required to achieve a real modification of the rheological behaviour of the bituminous mixture [3].

Nevertheless, crumb rubber can also be incorporated into SMA mixtures. It has been demonstrated that the use of crumb rubber to modify the binder makes the addition of cellulose fibres in SMA mixtures unnecessary, as the rubberized binder is not prone to drainage [4]. The modification of SMA and gap-graded asphalt mixtures with crumb rubber modifier (CRM) has been extensively studied. There is consensus on the fact that rubber modification improves resistance to plastic deformations [5,6]. It has also been shown that the incorporation of CRM into an asphalt mixture improves its modulus and its fatigue behaviour [7]. It has also been observed that the use of CRM bitumen in wearing layers has a sound-absorbing effect, reducing traffic noise pollution [8,9].

However, rubberized asphalt mixtures are usually produced at high temperatures due to the high viscosity of rubberized binders, which in turn results in higher energy consumption and greater production of greenhouse gases (GHG). The warm mix asphalt (WMA) technology can reduce the manufacturing and compaction temperatures, making working conditions better (especially at paving sites) and improving sustainability. This technology has been frequently used in mixtures without CRM in Europe [10] and worldwide [11,12]. In the case of modified CRM asphalt mixtures, the leading technologies involved in temperature reduction are organic and chemical additives [13], but the application of foamed bitumen technologies has not been frequent. Organic additives are usually waxes, with melting points around 110 °C, which a decrease in the viscosity of the CRM modified binder at the manufacturing and compaction temperatures.

Regarding the chemical additives, different types of WMA additives are reported in the literature. They are usually formed by a package of products such as surfactants, emulsification agents, aggregate coating enhancers and anti-stripping additives, and they are added to the binder during the manufacturing process. They do not reduce the viscosity of the binder, but favour binder–aggregate adhesion, through surface mechanisms that improve the coating.

As for the temperature reduction that can be achieved with these techniques, it depends on the additive used. When organic waxes are used, an equivalence study can be done to determine the temperature reduction. However, it is necessary to study the compactness of bituminous mixtures to verify that they are properly compacted. In the case of chemical additives, the previous viscosity study does not contribute anything, since these additives do not act on the viscosity of the binder.

In the review by Wang et al. [13], depending on the technology used, temperature reductions of between 20 and 30 °C were achieved in various test sections. Oliveira et al. [14] obtained temperature reductions of 30 °C in an SMA mixture with a surfactant additive. Through a study of the Marshall compaction curve, they determined this temperature reduction, and they verified that there was a slight worsening in the resistance to plastic deformation, while the rest of the properties remained approximately constant. Rodríguez-Alloza and Gallego [15,16] found that the working temperature of a gap-graded mixture with rubber-modified bitumen could be reduced by 30 °C by adding organic waxes. However, the indirect tension stress ratio (ITSR) decreased with respect to the values for the reference mixture. The compaction method was the Marshall one, and they admitted a 1% increase in the air void content. Akisetty et al. also admitted the same tolerance regarding the air void content [17], which also obtained a reduction of 30 °C in a dense-graded mixture with CRM and a wax, using a gyratory compactor. Other investigations have compared the results obtained by Marshall and gyratory compaction in SMA mixtures with CRM incorporating waxes or chemical additives [18,19]. The tests were performed for compaction temperatures of 160 and 144 °C. They observed that the rotating compaction allowed for a better distinction between the different additives and temperatures. When studying a dense-graded mixture made with CRM, Lee et al. [20] found that the study of gyratory compactability was more sensitive than the study with the Marshall compactor. However, in mixtures without rubber, both compactors were suitable.

One of the problems that has been observed when working with mixtures with CRM at reduced temperatures has been the worsening of resistance to water attack. This fact is perhaps related to the destabilization of the colloidal structure of the bitumen due to the absorption of oils by the CRM reported by Xu et al. [21]. The ITSR test has been the most used procedure to study the water sensitivity of warm mixtures without CRM [22] and has allowed us to establish that acidic aggregate is problematic and the reduction of temperature demands an antistripping additive to exceed the minimum ITSR values admitted in the specifications of hot mixes. For example, Khedmati et al. [23] used an antistripping additive together with a wax to improve the water resistance of a warm SMA mixture with siliceous aggregates, achieving a significant improvement in the ITSR values. For the manufacturing of the specimens, they used gyratory compaction. In another case study [24] of an SMA mixture with CRM and a WMA additive surfactant, which was diluted to 99% water, with basalts being added as aggregate and limestone as fine aggregate, the ITSR value dropped from 85% to 75% by reducing the working temperature from 180 to 160 °C. They compacted the specimens with a Marshall compactor.

With the previous results, it seems clear that the reduction of the ITSR may be influenced by the type of additive used, the magnitude of the temperature reduction and also by the method of compacting the specimens. However, in WMA mixtures with rubber, which can be difficult to compact, the influence of the compaction method is poorly studied. It should be borne in mind that each compaction procedure can lead to a different rearrangement of the particles. For example, gyratory compaction has a kneading effect that is not found in Marshall compaction. According to Vega-Zamanillo et al. [25], when studying dense-graded mixtures with WMA additives and without a rubber modifier, the ITSR values were better when the specimens were compacted with the gyratory compactor. Jiang et al. [26] also found that the Marshall compactor can lead to mechanical properties 20% lower than those of compacted specimens, up to the same density, with a vertical vibration plate. Several studies that have compared laboratory compaction procedures with real-scale compaction [27,28,29] have concluded that the gyratory compactor may represent field compaction better than other laboratory compactors.

Draft specifications for SMA mixtures are currently being prepared in Spain. One of the issues that are still being studied is the most appropriate compaction method for manufacturing ITSR specimens. Usually, these specimens are manufactured with the Marshall compactor. Marshall is known and used in all countries; however, laboratories around the world are moving towards the gyratory compactor [30].

Considering the information mentioned above, this research aimed to compare the Marshall and the gyratory compactors to determine the optimal procedure for studying the compactability and for the production of specimens for testing the water sensitivity (ITSR) of SMA mixtures made with CRM and a WMA additive of vegetable origin. This type of mixture can offer an excellent field to compare both methods since the CRM and the reduced temperature can make the mixture especially challenging to be compacted.

## 2. Materials and Methods

### 2.1. Materials

#### 2.1.1. Binders, Rubber, Polymer and WMA Additive

Two different virgin binders were used in this study: 50/70 and 160/220 penetration bitumens. The rubber was manufactured by mechanical grinding at ambient temperature. The gradation of crumb rubber is presented in Table 1.

The content of crumb rubber modifier was 10% over the weight of the binder. SBS (styrene-butadiene-styrene), an elastomeric compound, was also added to the binders at a proportion of 2.5% over the weight of the binder. The chemical additive tested to reduce the compaction temperatures was Nosbur^®^ ThErmo+. It is a vegetable liquid WMA additive with surfactant properties, is thermo-resistant and stable to store, and additionally improves the aggregate–binder adhesiveness. It is formulated with natural compounds of vegetal origin exempt from any hazard classification. The supplier recommends a dosage of 0.5% over the weight of the binder.

As the CRM binders usually show problems with storage stability, they are produced for specific projects. For this reason, the authors could not order a sample of this type of binder from an industry producer. The modified binder for this investigation was produced at the laboratory, according to the previous experience of the authors. As for the procedure to modify binders in the laboratory, a portion of 600 g of virgin bitumen was placed in an oil bath at 140 °C keeping temperature with a precision of ±1 °C. After this, the chemical additives were added to the bitumen and blended for 15 min at 900 rpm. The blend was heated up to 185 °C, then the rubber was added, and the sample was stirred for 45 min at 4000 rpm. After that, the SBS polymer was added, the binder was stirred for 15 min at 4000 rpm and, finally, the temperature was set at 165 °C and the binder mixed at 900 rpm for 15 min. This was then incorporated into the manufacture of SMA mixtures. The composition of the modified binders in this study was based on a standard formulation to produce a PMB 35/80-65C with a range of 45–80 × 10^−1^ mm for penetration (EN-1426) and a ring and ball temperature equal or higher than 65 °C. It is commonplace to prepare first a base bitumen composed of 50/70 × 10^−1^ mm penetration and 160/200 × 10^−1^ mm penetration. In this particular case, the proportion was 50%–50%. Afterwards, the additives, rubber and SBS polymer were added. Table 2 presents a basic characterization of the softening point (EN 1427), penetration (EN 1426) and elastic recovery (EN 13398) of the virgin and modified binders in this research.

#### 2.1.2. Aggregates

**Figure 1:**
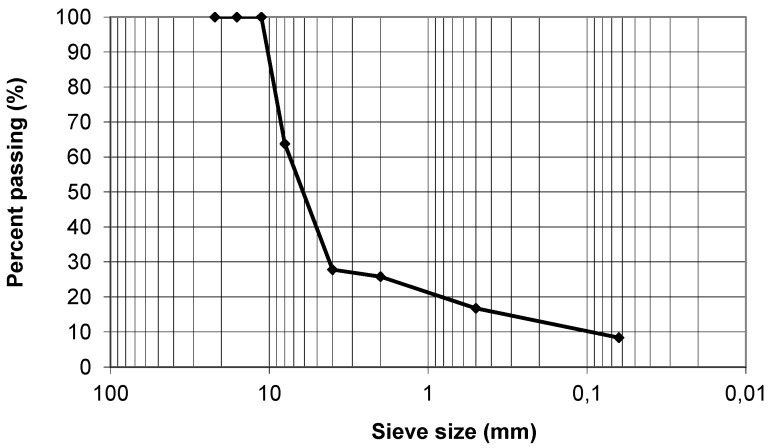
Grading curve of the mixtures.

The following aggregates were used to produce the rubber-modified SMA mixtures (RSMA); ophite as a coarse aggregate, limestone as a fine aggregate and calcareous filler. The different fractions and percentages can be seen in Table 3 and the grading curve in Figure 1.

#### 2.1.3. RSMA Control Mixture and W-RSMA Mixture

In this study, two different bituminous mixtures were produced: RSMA (the control mixture) and warm RSMA mixtures (W-RSMA), produced at different temperatures. All of them contained 6.2% of modified bitumen over the weight of aggregates. A list of the mixtures under study is presented in Table 4. The binder name is included as well as the content of rubber, SBS and WMA additive and the temperatures selected for the production of the mixtures.

The production temperature of the RSMA control mixture was the usual temperature for this type of mixture (165 °C). The production temperatures of W-RSMA mixtures were the same as the control mixture (165 °C) and, to assess the efficacy of the chemical additive, the production temperatures were also lowered to 155 °C, 145 °C and 135 °C.

### 2.2. Test Methods

#### 2.2.1. Compactability

The tests were carried out according to the European standard EN 12697-10. This standard considers two test procedures: impact or gyratory compaction. With the impact compaction, samples were prepared at the selected temperatures and compacted using the Marshall compactor following EN 12697-30 (Specimen preparation by impact compactor), with up to 100 blows on each side. The variation in the sample thickness was monitored and recorded during the compaction operation. Four samples were tested for every mixture under study. On the other hand, with the gyratory compaction (EN 12697-31, Specimen preparation by gyratory compactor) the variation of the density of the asphalt mixture is plotted as a function of the number of rotations. The parameters of the compactor were a vertical pressure application of 600 kPa, a compaction angle of 0.82° and a speed of 30 rpm).

Three specimens for each temperature were tested to obtain an average densification curve. In both the Marshall and gyratory compaction, the average densification curves at different temperatures were compared later in order to determine the theoretical attainable reduction. In this paper, the nomenclature of a mixture refers to its production or mixing temperature. Nevertheless, in all cases, the compaction temperatures were established as 10 °C below the production temperature in order to consider the loss of temperature during transportation of the mixture from the asphalt plant to the worksite.

#### 2.2.2. Water Sensitivity 

The water sensitivity of the asphalt mixtures was determined according to EN 12697-12 (Water sensitivity of bituminous specimens). This procedure is the most used to study the resistance to water attack in asphalt mixtures [22]. A series of specimens were prepared with the Marshall impact compactor and a second series with the gyratory compactor. Regardless of the method of compaction, four dry specimens were set aside at room temperature (20 °C) while four wet samples were placed in a thermostatic water bath for three days at 40 °C. After this period, all the specimens were placed in a thermostatic bath at 15 °C for two hours before subjecting them to the indirect tensile strength test (ITS), according to EN 12697-23. Finally, to analyse the water sensitivity, the indirect tension stress ratio (ITSR) between the average strength results of the wet (ITS_w_) and dry (ITS_d_) specimens was calculated. In all cases, the maximum density (EN 12697-5), the bulk density (EN-12697-6) and the air void content by the dry saturated surface method (EN12697-8) were determined.

## 3. Results and Discussion

### 3.1. Compactability with Marshall Compactor

**Figure 2:**
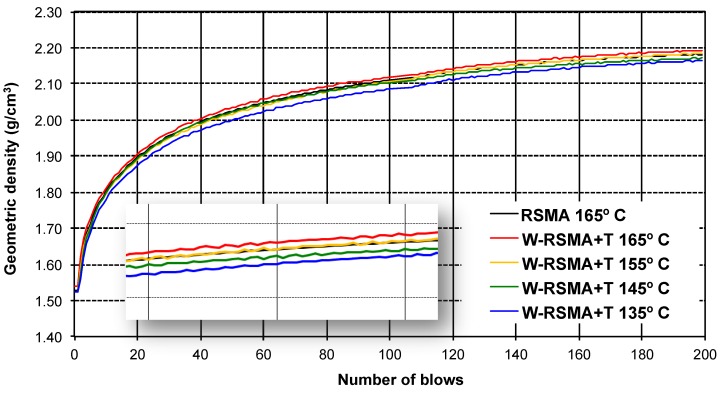
Compactability with Marshall compactor and Nosbur^®^ ThErmo+.

The compactability curves with the Marshall compactor for the RSMA control mixture at 165 °C and the W-RSMA mixtures at different temperatures are presented in Figure 2. They include a magnification of the curves for more precise observation, and the possible reduction of the production temperatures when the additives are incorporated can be estimated. The production temperatures of the W-RSMA mixtures correspond to the temperature that achieves a densification curve as close as possible to the reference RSMA 165 °C curve.

It can be noted that, as the production temperature of the W-RSMA mixtures was reduced, the density of the mixtures decreased and the curve of the control mixture (RSMA 165 °C) and the curve with W-RSMA+T 155 °C overlapped, meaning a possible reduction of approximately 10 °C. It must be highlighted that this is a minor reduction in temperature. It seems that the vegetable additive is not efficient or that the Marshall method is not able to properly assess its effect on the compactability of the asphalt mixture.

### 3.2. Compactability with Gyratory Compactor

**Figure 3:**
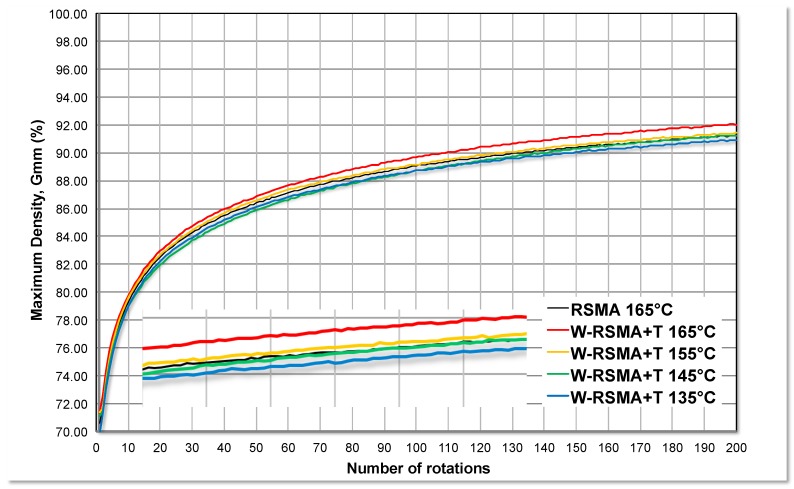
Compactability with the gyratory compactor and Nosbur^®^ ThErmo+.

According to Lee et al. [20], the Marshall compactor seems to be less sensitive to temperature reduction than the gyratory compactor. Therefore, the compactability study was repeated using the gyratory compactor. Figure 3 presents the compactability curves. Using this method, the variation of the percentage of maximum density (%G_mm_) and the corresponding number of rotations were obtained. A magnification of the final section of the densification curves is included in the figures so the curves can be analysed in detail and an estimation of the suitable reduction can be made. For the W-RSMA mixtures with Nosbur^®^ ThErmo+ (Figure 3), the attainable reduction of the production temperature is around 20 °C. It may be noted that the curve of the control mixture (RSMA 165 °C) and the mixture curve produced at 145 °C (W-RSMA+T 145 °C) almost overlapped.

### 3.3. Water Sensitivity with Marshall Compactor Samples

The air void contents of the samples prepared with the Marshall compactor with 50 blows/side are shown in Table 5. 

To verify that the average of the voids in the specimens of each group were different, a single-factor analysis of variance was performed (the type of asphalt mix). For a significance level of 95%, the F statistic had a value of 57.71759259, higher than the critical F value 2.641465186, which indicates that the null hypothesis of the equality of the means of all the groups is false, and, therefore, the groups have different means.

**Figure 4:**
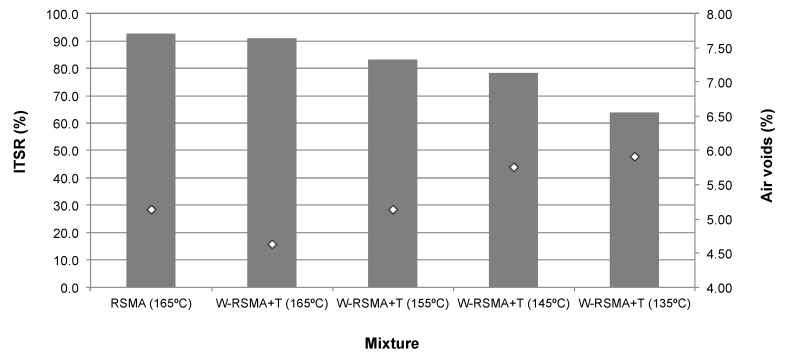
ITSR results for samples prepared with Marshall compactor.

The ITS and the ITSR results are shown in Table 6, as well as in Figure 4. It can be observed that when the WMA additive is added, the ITS results are slightly worse and ITSR values lowered as the production temperatures decreased.

In terms of specifications to be accomplished, it can be observed in Figure 4 that when the compaction temperature of the W-RSMA mixtures is the same as the control mixture (RSMA 165 °C), the ITSR value is very similar and above the 90% required for the surface layer. Nevertheless, as the compaction temperatures decrease, the ITSR values worsen. Even mixtures produced at 155 °C do not fulfil the 90% requirement for the water sensitivity of a surface layer in Spain [31]. This worsening of the water sensitivity can be related to the increase in the number of air voids in the mixture, as presented in Figure 4. The lack of efficiency of the WMA additives with the Marshall compactor results in high void content, making the mixtures more vulnerable to water attack. These results achieved with specimens compacted by the Marshall hammer mean that, regarding water sensitivity, no reduction of the production temperatures of the mixtures are recommended.

### 3.4. Water Sensitivity with Gyratory Compactor

As the resistance to water attack in specimens produced by the Marshall compactor showed poor results, it was decided to compact the specimens for this test with the gyratory compactor, according to Vega-Zaramillo et al. [25]. The use of the gyratory compactor for rubberized hot mixtures has been also recommended by Tarefder et al. [28]. The results for the bulk density and the air void contents of the specimens are shown in Table 7.

To determine whether the average values for the air void content in each mixture were different from each other, an analysis of variance of a factor (type of mixture) and for a significance level of 95% was performed. A statistic value F = 39.86452242 was obtained, higher than the critical F value = 2.641465186, which showed that the means of the groups are different from the average of all the test specimens under study. Therefore, depending on the type of mixture, the specimens were compacted differently.

**Figure 5:**
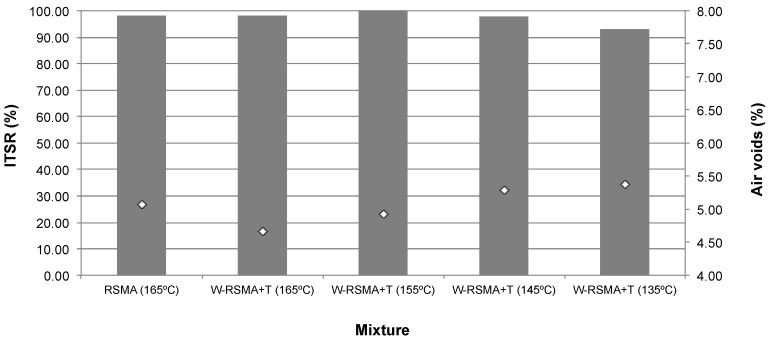
ITSR results for samples prepared with the gyratory compactor.

The ITS results for the specimens prepared with 120 rotations with the gyratory compactor are shown in Table 8 and Figure 5. This level of compaction was selected to achieve an air void content in the control RSMA mixture (5.07 g/cm^3^, Table 7) similar to that achieved by 50 blows/side with the Marshall compactor (5.13 g/cm^3^, Table 5), in both cases at 165 °C.

When the additives are incorporated into mixtures produced at 165 °C, which is the production temperature of the control mixture, they present similar ITSR values to those of the control mixture (Figure 5). Even at lower temperatures (155 °C–135 °C) the ITSR values are similar to that of the control mixture and always above the 90% required for surface layers, according to the PG-3 in the Spanish draft specifications for SMA mixtures [31].

Regarding the air void content (Figure 5), a higher air void content is not noticeable when the temperature decreases. So, in terms of resistance to water attack, the production temperature can be reduced by 30 °C. From these results, it is evident that not only does the WMA technology affect the water sensitivity of the mixtures, as stated by Xu et al. [22]. The compact method is highly influential; the kneading effect of the gyratory compactor can achieve a lower air void content than the Marshall impact compactor when reducing temperatures with the WMA additive.

However, the difference in air void content between the specimens produced with the Marshall hammer and the gyratory compactor is around 1%, while the ITSR values range from 63% to 93%. Further investigation is needed to confirm that the kneading effect, apart from improving the efficiency of densification (and preventing the entrance of water) is able to enhance the binder–aggregates adhesiveness, as suggested in previous investigations [25,26].

### 3.5. Attainable Reduction of Production Temperature

According to the results of Section 3.1, Section 3.2, Section 3.3 and Section 3.4, it would be possible to reduce the production temperatures of the mixtures under study, thanks to the use of the WMA additive Nosbur^®^ ThErmo+. However, this reduction depends on the property analysed and the method of compaction. In the case of the Marshall compactor, no reduction of the production temperature would be recommended as the water sensitivity falls below the minimum values admitted for wearing courses. Nevertheless, considering the results obtained in the mixtures prepared with the gyratory compactor, a temperature reduction of 20 °C is viable with the additive Nosbur^®^ ThErmo+.

## 4. Conclusions

The objective of this research was to determine the most appropriate type of compaction for the study of SMA mixtures. Currently, in Spain, there is only a draft of specifications for SMA mixtures that establishes the Marshall compaction method. However, some researchers have reported that the gyratory compaction method is more effective. 

To help clarify this issue, this study evaluated the effects of a chemical warm mix asphalt (WMA) additive (Nosbur^®^ ThErmo+) on the workability of a rubberized stone mastic asphalt (RSMA) mixture. The tests performed were for compactability, evaluated by two different procedures (impact and gyratory compaction), and water sensitivity.

The binder content of the SMA control mixture was 6.2% modified bitumen over the weight of aggregates, including 10% rubber and 2.5% SBS over the weight of net bitumen. The RSMA mixtures with WMA additive (W-RSMA mixtures) also incorporated Nosbur^®^ ThErmo+ (0.5% over the weight of the binder). The control mixture was produced at 165 °C and the W-RSMA mixtures were prepared at 165 °C, 155 °C, 145 °C and 135 °C. From the results of the tests performed, the following conclusions can be drawn.

Depending on the procedure used to perform the compactability tests (impact or gyratory), different results were obtained, and different attainable reductions of compaction temperatures resulted. It seems that gyratory compaction is more capable of distinguishing the presence of the WMA additive compared to the Marshall procedure. When using the Marshall procedure, the working temperature can be reduced by only 10 °C by adding Nosbur^®^ ThErmo+. Meanwhile, with the gyratory compactor, a reduction of 20 °C is attainable by adding this additive. 

Regarding water sensitivity, when the samples were prepared with the Marshall compactor, the indirect tension stress ratio (ITSR) did not fulfil the water sensitivity requirements in Spain (90%), except for the mixtures prepared at 165 °C (the same production temperature as the control mixture). Therefore, no temperature reduction is recommended, even with the presence of the WMA additive. 

However, when the samples were prepared with the gyratory compactor, the ITSR values of the mixtures at lower temperatures remained above the 90% required. Bearing in mind that most of the authors in the literature review reported the good correspondence of the gyratory compacted specimens with the field cores, this result seems to indicate that the reduction of the temperature by using the tested WMA additive could reach 20 °C.

Above everything, the result of this investigation is that the gyratory compactor is preferable to the Marshall test to design and test SMA mixtures, especially when they are made with CRM and WMA additives.

## Figures and Tables

**Table 1:** Gradation of the crumb rubber modifier (CRM).

Sieve (mm) (UNE 933-2)	Passing (%)
2	100
1.5	100
1	100
0.5	94.1
0.25	23.7
0.125	3.7
0.063	0.4

**Table 2:** Binders name, composition and basic characterization.

Binder Name for This Study	Binders Composition	Softening Point T (°C) EN 1427	Penetration (10^−1^ mm) EN 1426	Elastic Recovery (%) EN 13398
B	50% 50/70 + 50% 160/220	43.2	98.0	2.0
B+2.5S+10R	50% 50/70 + 50% 160/220 + 0.5% Nosbur^®^ ThErmo+ 10% rubber + 2.5% SBS	66.6	55.0	83.0

**Table 3:** Aggregates.

Aggregate	Fraction (mm)	Proportion (%)
Ophite	8/11	36.2
Ophite	4/8	36.0
Limestone	2/4	2.0
Limestone	0.5/2	9.0
Limestone	0.25/0.5	4.0
Limestone	0.063/0.25	4.0
Calcareous filler		8.8

**Table 4:** Control mixture and stone mastic asphalt with warm mix asphalt additives.

Mixture	Binder	SBS (%)	CRM (%)	Additive (%)	WMA Additive	Production T^a^ (°C)
RSMA	B + 2.5 S + 10R	2.5	10	0	−	165
W-RSMA + T	B + 0.5T + 10R + 2.5S	2.5	10	0.5	Nosbur^®^ ThErmo+	165, 155, 145, 135

**Table 5:** Volumetric characteristics of the mixtures.

	Specimen	Bulk Density (g/cm^3^)		Air Void (%)	
RSMA 165 °C	#1	2.295	Av = 2.295 SD = 0.0052	5.13	Av = 5.13 SD = 0.213
	#2	2.295		5.13	
	#3	2.289		5.37	
	#4	2.298		5.00	
	#5	2.304		4.75	
	#6	2.289		5.37	
	#7	2.291		5.29	
	#8	2.298		5.00	
W-RSMA+T 165 °C	#1	2.305	AV = 2.307 SD = 0.0053	4.71	Av = 4.63 SD = 0.29
	#2	2.314		4.34	
	#3	2.299		4.96	
	#4	2.312		4.42	
	#5	2.307		4.63	
	#6	2.313		4.38	
	#7	2.303		4.80	
	#8	2.305		4.71	
W-RSMA+T 155 °C	#1	2.289	Av = 2.295 SD = 0.0041	5.37	Av = 5.13 SD = 0.170
	#2	2.296		5.08	
	#3	2.296		5.08	
	#4	2.295		5.13	
	#5	2.295		5.13	
	#6	2.298		5.00	
	#7	2.289		5.37	
	#8	2.301		4.88	
W-RSMA+T 145 °C	#1	2.276	Av = 2.280 SD = 0.007	5.91	Av = 5.75 SD = 0.196
	#2	2.284		5.58	
	#3	2.283		5.62	
	#4	2.273		6.04	
	#5	2.284		5.58	
	#6	2.279		5.79	
	#7	2.275		5.95	
	#8	2.285		5.54	
W-RSMA+T 135 °C	#1	2.272	Av = 2.276 SD = 0.0061	6.08	Av = 5.91 SD = 0.173
	#2	2.274		5.99	
	#3	2.282		5.66	
	#4	2.278		5.83	
	#5	2.279		5.79	
	#6	2.275		5.95	
	#7	2.269		6.20	
	#8	2.278		5.83	

**Table 6:** Results of water sensitivity test for the mixtures prepared with the Marshall compactor.

Mixture	Production T^a^ (°C)	ITSd (MPa) EN 12697-23	ITSw (MPa) EN 12697-23	ITSR (%) EN 12697-12
RSMA	165	2.129	1.975	92.8
	165	1.858	1.687	90.8
W-RSMA + T	155	1.915	1.591	83.1
	145	1.785	1.396	78.2
	135	1.954	1.248	63.9

**Table 7:** Volumetric characteristics of the mixtures.

	Specimen	Bulk Density (g/cm^3^)		Air Void (%)	
RSMA 165 °C	#1	2.298	Av = 2.296 SD = 0.003	5.00206697	Av = 5.07 SD = 0.11
	#2	2.292		5.250103348	
	#3	2.294		5.167424556	
	#4	2.299		4.960727573	
	#5	2.3		4.919388177	
	#6	2.295		5.126085159	
	#7	2.295		5.126085159	
	#8	2.297		5.043406366	
W-RSMA + T 165 °C	#1	2.303	AV = 2.306 SD = 0.004	4.795369988	Av = 4.67 SD = 0.154
	#2	2.309		4.547333609	
	#3	2.311		4.464654816	
	#4	2.307		4.630012402	
	#5	2.308		4.588673005	
	#6	2.299		4.960727573	
	#7	2.306		4.671351798	
	#8	2.307		4.630012402	
W-RSMA + T 155 °C	#1	2.301	Av = 2.300 SD = 0.003	4.87804878	Av = 4.92 SD = 0.128
	#2	2.303		4.795369988	
	#3	2.298		5.00206697	
	#4	2.303		4.795369988	
	#5	2.303		4.795369988	
	#6	2.299		4.960727573	
	#7	2.295		5.126085159	
	#8	2.297		5.043406366	
W-RSMA + T 145 °C	#1	2.294	Av = 2.391 SD = 0.002	5.167424556	Av = 5.29 SD = 0.100
	#2	2.292		5.250103348	
	#3	2.287		5.456800331	
	#4	2.291		5.291442745	
	#5	2.294		5.167424556	
	#6	2.29		5.332782141	
	#7	2.289		5.374121538	
	#8	2.292		5.250103348	
W-RSMA + T 135 °C	#1	2.287	Av = 2.289 SD = 0.003	5.456800331	Av = 5.35 SD = 0.125
	#2	2.292		5.250103348	
	#3	2.285		5.539479124	
	#4	2.289		5.374121538	
	#5	2.292		5.250103348	
	#6	2.287		5.456800331	
	#7	2.293		5.208763952	
	#8	2.292		5.250103348	

**Table 8:** Results of water sensitivity test for the mixtures prepared with the gyratory compactor.

Mixture	Production T^a^ (°C)	ITSd (MPa) EN 12697-23	ITSw (MPa) EN 12697-23	ITSR (%) EN 12697-12
RSMA	165	2.04	2.082	98.0
	165	1.962	1.923	98.0
W-RSMA + T	155	1.845	1.826	99.9
	145	1.886	1.843	97.7
	135	1.819	1.692	93.0
